# Supplementary material for: Restoring a nearly free-standing character of graphene on Ru(0001) by oxygen intercalation
Source: Sci Rep. 2016 Feb 8;6:20285. doi: 10.1038/srep20285 (PMC4745051; doi:10.1038/srep20285)
Supplement: Supplementary Information [file srep20285-s1.pdf]

Supplementary material for manuscript:

Restoring a nearly free-standing character of graphene on Ru(0001) by oxygen intercalation

Elena Voloshina,[1] Nikolai Berdunov,[2] and Yuriy Dedkov[2]

[1]Humboldt-Universität zu Berlin, Institut für Chemie, 10099 Berlin, Germany

[2]SPECS Surface Nano Analysis GmbH, Voltastraße 5, 13355 Berlin, Germany

List of figures:

Fig. S1. (a) Top and side view of the graphene/1ML O/Ru(0001) obtained after the structural optimization. The high-symmetry places in (a) are marked by circle, rhombus, star, and triangle for ATOP, FCC, BRIDGE, and HCP positions, respectively. In (b) the structure of the respective system is overlaid with the calculated difference electron density, $\Delta\rho_{\mathrm{gr/O/Ru}}(r) = \rho_{\mathrm{gr/O/Ru}}(r) - (\rho_{\mathrm{gr}}(r) + \rho_{\mathrm{O/Ru}}(r))$, plotted in units of $e/\mathrm{\AA}^3$. (c) Carbon-site projected density of states calculated for all high-symmetry places of graphene/1ML O/Ru(0001). Greyed plot in (c) shows the corresponding DOS for the free-standing graphene.

Fig. S2. The height variation of the carbon atoms in the graphene layer on (a) Ru(0001), (b) 0.5 ML-O-(2×1)/Ru(0001), and (c) 1 ML-O/Ru(0001). The colour bar on the left-hand side represents the hight scale in Å.

Fig. S3. (a) Carbon-projected DOSs for different high-symmetry places of the graphene/Ru(0001) system. (b) Ru-projected DOS for graphene/Ru(0001).

Fig. S4. (a) Carbon-projected DOSs for different high-symmetry places of the graphene/0.5 ML-O-(2×1)/Ru(0001) system. (b) Oxygen-projected DOS for graphene/0.5 ML-O-(2×1)/Ru(0001). (c) DOS projected onto Ru-atoms of the topmost metal-layer for graphene/0.5 ML-O-(2×1)/Ru(0001).

Fig. S5. Calculated CC STM images of (a) graphene/Ru(0001), (b) graphene/0.5 ML-O-(2×1)/Ru(0001), and (c) graphene/1 ML-O/Ru(0001) obtained at 300 meV of the bias voltage.

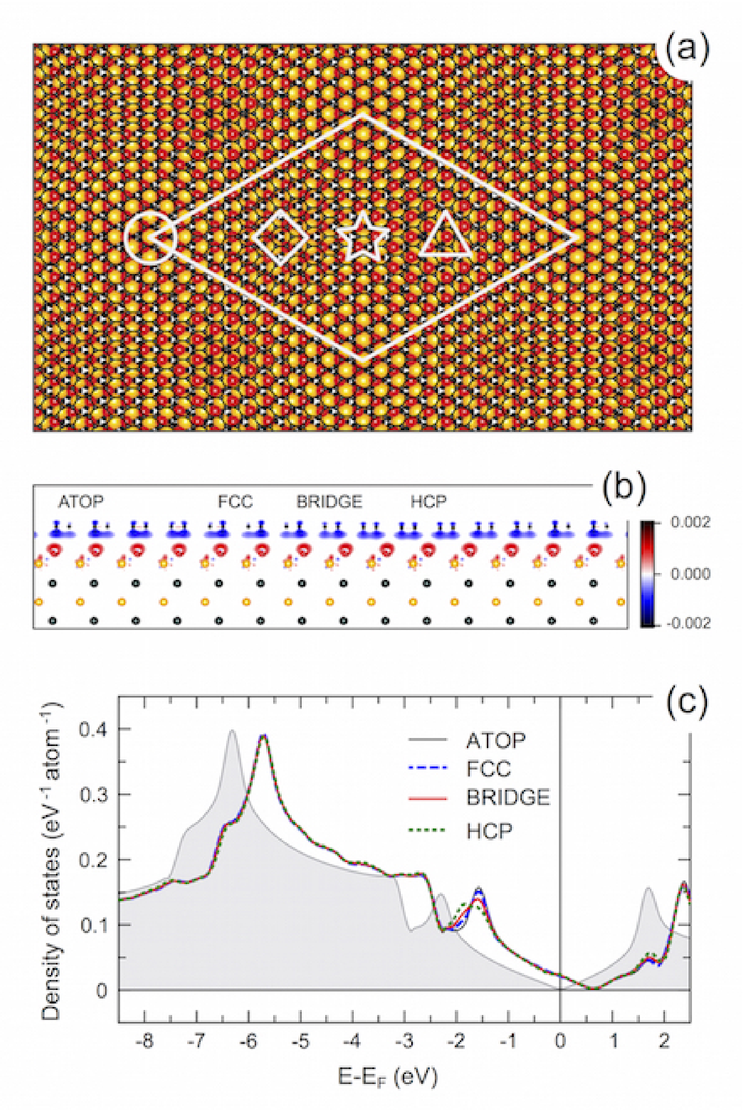

Fig. S1. (a) Top and side view of the graphene/1ML O/Ru(0001) obtained after the structural optimization. The high-symmetry places in (a) are marked by circle, rhombus, star, and triangle for ATOP, FCC, BRIDGE, and HCP positions, respectively. In (b) the structure of the respective system is overlaid with the calculated difference electron density, $\Delta\rho_{\mathrm{gr/O/Ru}}(r) = \rho_{\mathrm{gr/O/Ru}}(r) - (\rho_{\mathrm{gr}}(r) + \rho_{\mathrm{O/Ru}}(r))$, plotted in units of $e/\text{Å}^3$. (c) Carbon-site projected density of states calculated for all high-symmetry places of graphene/1ML O/Ru(0001). Greyed plot in (c) shows the corresponding DOS for the free-standing graphene.

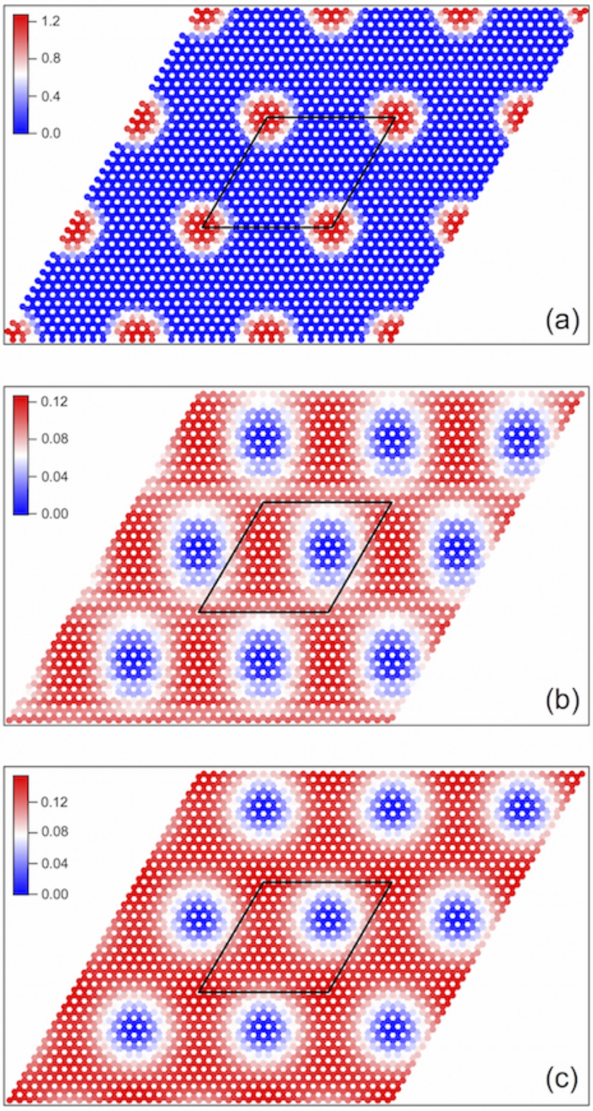

Fig. S2. The height variation of the carbon atoms in the graphene layer on (a) Ru(0001), (b) 0.5 ML-O-(2 × 1)/Ru(0001), and (c) 1 ML-O/Ru(0001). The colour bar on the left-hand side represents the hight scale in Å.

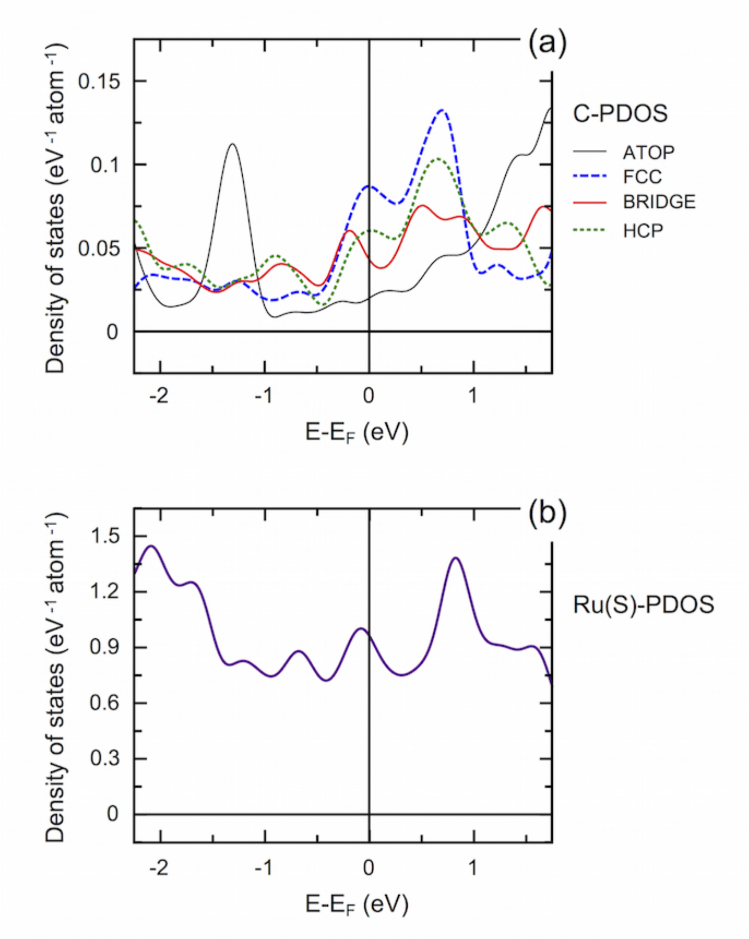

Fig. S3. (a) Carbon-projected DOSs for different high-symmetry places of the graphene/Ru(0001) system. (b) Ru-projected DOS for graphene/Ru(0001).

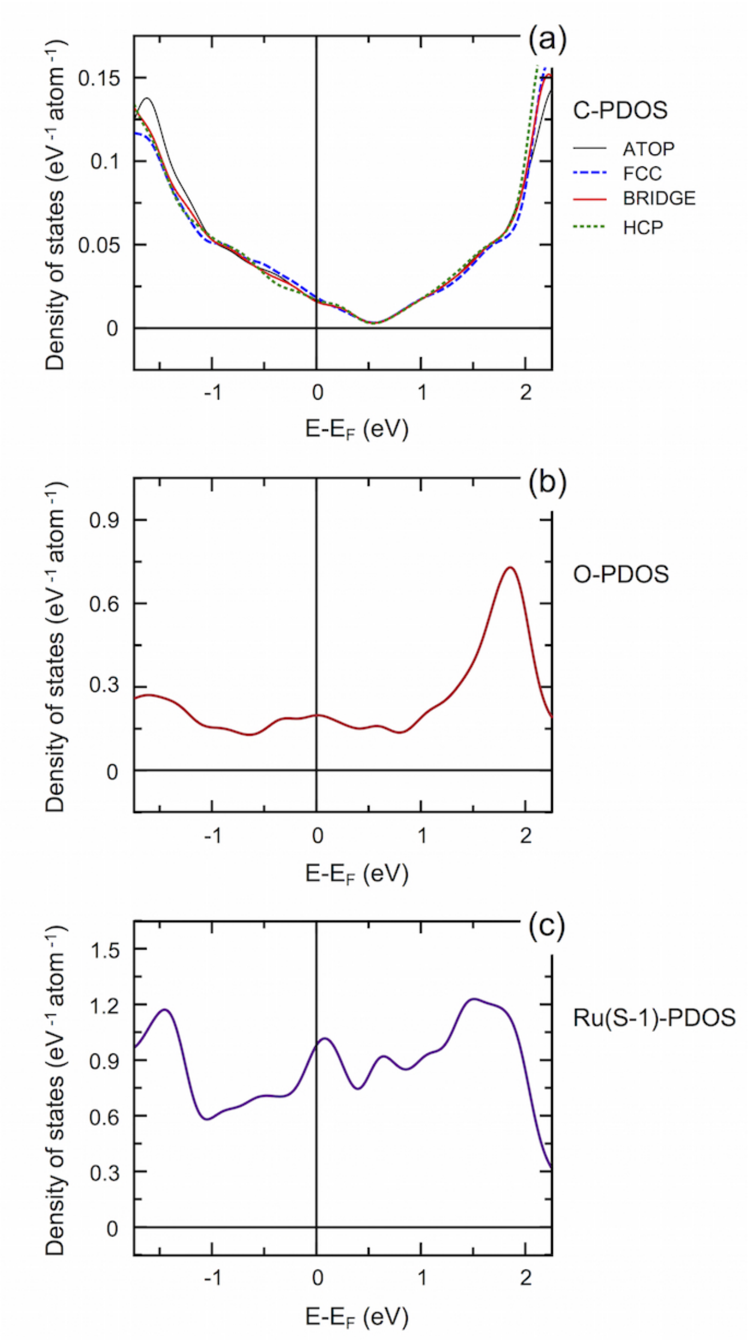

Fig. S4. (a) Carbon-projected DOSs for different high-symmetry places of the graphene/0.5 ML-O-(2 × 1)/Ru(0001) system. (b) Oxygen-projected DOS for graphene/0.5 ML-O-(2 × 1)/Ru(0001). (c) DOS projected onto Ru-atoms of the topmost metal-layer for graphene/0.5 ML-O-(2 × 1)/Ru(0001).

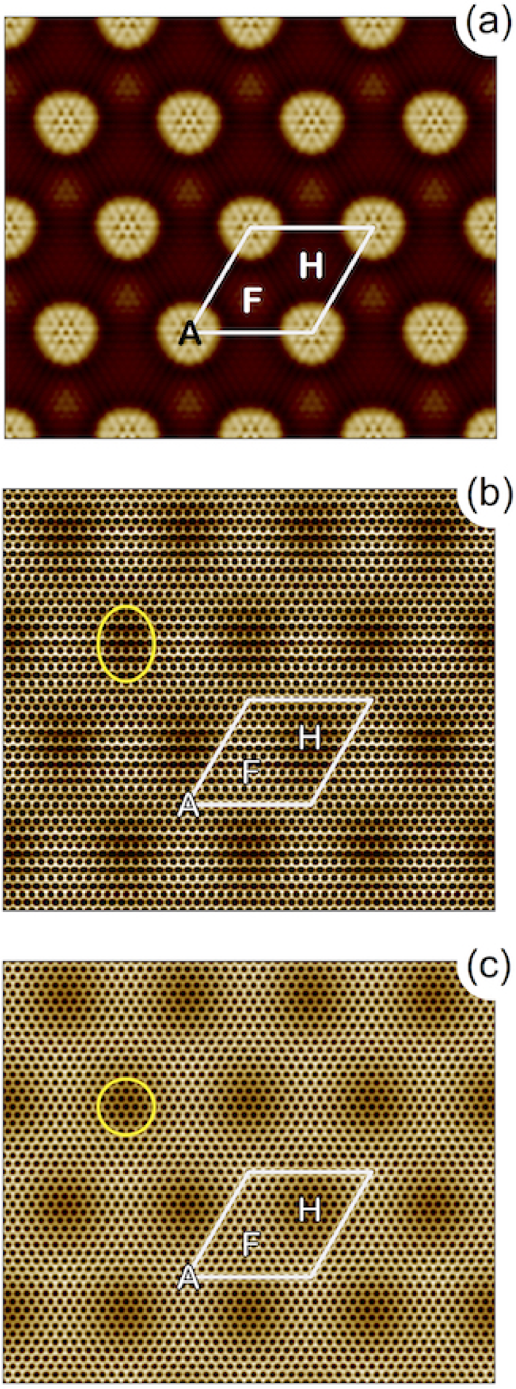

Fig. S5. Calculated CC STM images of (a) graphene/Ru(0001), (b) graphene/0.5 ML-O-(2 × 1)/Ru(0001), and (c) graphene/1 ML-O/Ru(0001) obtained at 300 meV of the bias voltage.
